# Preparation and evaluation of novel mixed micelles as nanocarriers for intravenous delivery of propofol

**DOI:** 10.1186/1556-276X-6-275

**Published:** 2011-03-31

**Authors:** Xinru Li, Yanhui Zhang, Yating Fan, Yanxia Zhou, Xiaoning Wang, Chao Fan, Yan Liu, Qiang Zhang

**Affiliations:** 1 Department of Pharmaceutics, School of Pharmaceutical Sciences, Peking University, Xueyuan Road 38, Haidian District, Beijing 100191, People's Republic of China

## Abstract

Novel mixed polymeric micelles formed from biocompatible polymers, poly(ethylene glycol)-poly(lactide) (mPEG-PLA) and polyoxyethylene-660-12-hydroxy stearate (Solutol HS15), were fabricated and used as a nanocarrier for solubilizing poorly soluble anesthetic drug propofol. The solubilization of propofol by the mixed micelles was more efficient than those made of mPEG-PLA alone. Micelles with the optimized composition of mPEG-PLA/Solutol HS15/propofol = 10/1/5 by weight had particle size of about 101 nm with narrow distribution (polydispersity index of about 0.12). Stability analysis of the mixed micelles in bovine serum albumin (BSA) solution indicated that the diblock copolymer mPEG efficiently protected the BSA adsorption on the mixed micelles because the hydrophobic groups of the copolymer were efficiently screened by mPEG, and propofol-loaded mixed micelles were stable upon storage for at least 6 months. The content of free propofol in the aqueous phase for mixed micelles was lower by 74% than that for the commercial lipid emulsion. No significant differences in times to unconsciousness and recovery of righting reflex were observed between mixed micelles and commercial lipid formulation. The pharmacological effect may serve as pharmaceutical nanocarriers with improved solubilization capacity for poorly soluble drugs.

## Introduction

Propofol, chemically named 2,6-diisopropylphenol, is a highly effective and rapid intravenous anesthetics, which has gained increasing popularity in anesthesia in clinic. Its greatest advantage is the rapid recovery, even after long periods of anesthesia. A particularly low incidence of postoperative nausea and vomiting was also observed [1,2]. However, it also has some drawbacks such as poor water miscibility (150 μg/L) [3] and high lipophilicity (log*P *= 4.16) [4]. As vehicles for clinical delivery of anesthetics should be devoid of sedative and anesthetic properties, as well as toxic side effects, nearly all small-molecular weight organic solvents into which propofol is freely miscible are not useful. Therefore, propofol was initially formulated as a 1% solution in 16% Cremophor EL, which has been reported to induce undesirable side effects, such as the high occurrence of pain on injection and the risk of anaphylactic reactions associated to Cremophor EL. In view of the clinical importance of propofol, the alternative formulations such as oil/water emulsion consisting of soya bean oil, glycerol, and egg phosphatide (Diprivan^®^, Zeneca, UK) [5], microemulsions [6], inclusion complex [1], and polymeric micelles [7,8], have been developed to improve its solubility. Unfortunately, lipid-based emulsions suffer from other limitations including poor physical stability and the potential for embolism. Strictly aseptic techniques must be maintained when handling these formulations since they do not contain antimicrobial preservatives, and the vehicle can support rapid growth of microorganisms [9,10]. Among other particulate vehicles, polymeric micelles have presented their great potential in solubilization of poorly water-soluble drugs in recent years [11-14]. Generally, block copolymers with concentration above the critical micellization concentration (CMC) self-assemble into spherical polymeric micelles with a core-shell structure in water: the hydrophobic segments aggregate to form an inner core being able to accommodate hydrophobic drugs with improved solubility by hydrophobic interactions; the hydrophilic shell consists of a brush-like protective corona that stabilizes the micelles in aqueous solution [15-17]. Polymeric micelles as novel drug vehicles present numerous advantages, such as reduced side effects of drugs, selective targeting, stable storage, and stability toward dilution [17,18]. Furthermore, polymeric micelles possess a nanoscaled size with a narrow distribution. They can protect drugs against oxidation *in vitro *and premature degradation *in vivo *owing to their core-shell architecture [19,20]. More importantly, polymeric micelles are fabricated according to the physicochemical properties of drugs and the compatibility between the core of micelles and drug molecules [14,21]. Unfavorably, propofol-loaded polymeric micelles formed from poly(*N*-vinyl-2-pyrrolidone)-block-poly(d,l-lactide) (PVP-PLA) copolymers [7,8], the drug loading content (LC) was extremely low (7 to 12%). Therefore, there has been an urgent quest to develop an ideal polymeric micelles formulation that can solubilize propofol efficiently and solve some of the aforementioned problems.

This study presented a newly mixed micelle structure of methoxy poly(ethylene glycol)-b-poly(d,l-lactide) (mPEG-PLA) diblock copolymers and Solutol HS 15, which was expected to manifest increased drug loading efficiency, superior to those of the individual components, and at least maintain the efficacy of propofol. Solutol HS 15, the main component of which is the polyethylene glycol 660 ester of 12-hydroxy stearic acid, is a low molecular weight surfactant and recommended as non-ionic solubilizing agent to be added to injection solutions [22]. Its relatively bulky lipophilic portion might allow it for better drug solubilization. This micellar structure involved not only the modification of particles to hide the inner structure to prevent recognition by the physiological system, but also a new means of preparing micelles with higher drug LC from a copolymer with a low molecular weight surfactant. Therefore, the micelle preparation, propofol solubilization, and *in vitro *micelle properties were investigated by the size measurement, drug LC, encapsulation efficiency (EE), physical stability, and *in vitro *drug release. The *in vivo *pharmacological effect of propofol-loaded mixed micelles was also evaluated.

## Materials and methods

### Materials

Propofol was purchased from Zhongke Taidou Chemical Co., Ltd. (Shandong, China). mPEG5000-PLA4800 copolymers were synthesized in our laboratory as previously described [23]. Solutol HS15 was obtained from Yunhong Chemical Co., Ltd (Shanghai, China). The commercial lipid emulsion (CLE) injection for propofol (1%, w/v) was provided by Guorui Pharmaceutical Co., Ltd. (Sichuan, China). All other reagents were of analytical grade, except those for HPLC assay which were of HPLC grade.

### Animals

Sprague-Dawley (SD) rats weighing 200 ± 20 g were obtained from Animals Center of Peking University Health Science Center. All animals were provided with standard food and water *ad libitum *and were exposed to alternating 12-h periods of light and darkness. Temperature and relative humidity were maintained at 25°C and 50%, respectively. All care and handling of animals were performed with the approval of Institutional Authority for Laboratory Animal Care of Peking University.

### Determination of critical micellization concentration

The CMC was determined by fluorescence spectroscopy using pyrene (Fluka, > 99%, St. Louis, USA) as a hydrophobic probe as previously reported [11]. The fluorescence spectra of pyrene were measured at varying mixed micelle concentrations using a Shimadzu RF-5301 PC fluorescence spectrometer (Kyoto, Japan) at 25°C. The excitation wavelength was adjusted to 392 nm, and the detection of fluorescence was performed at 333 and 335 nm. CMC was measured from the onset of a rise in the intensity ratio of peak at 335 nm to peak at 333 nm in the fluorescence spectra of pyrene plotted versus the logarithm of polymer concentration.

### Preparation of mixed micelles

The drug-free mixed micelles were prepared by rotary evaporation method [23]. In brief, a methanol solution of mPEG-PLA and Solutol HS15 with different molar ratios was evaporated under vacuum at 60°C to form a homogeneous film. The resulting film was dispersed in 10 mL of water at 60°C and then vortexed for 3 min. Then the mixture was filtered through a 0.45-μm filter (Millex-GV, Millipore, USA) to obtain a clear and homogeneous micelle solution. The propofol-loaded mixed micelles were prepared by mixing propofol and the prepared drug-free micelle solution under magnetic agitation at room temperature. Then the resultant mixture was incubated at 4°C for 30 min and filtered. The filtrate was filled into ampoules and sealed under nitrogen.

### Particle size measurement

A certain volume of micelle solution was diluted with water to a definite volume in a flask and shaken gently to mix thoroughly. Samples were then passed through 0.22-μm pore-size filter before size measurement to remove dust particles. The average particle size and size distribution of micelles were determined by dynamic light scattering (DLS) (Zetasizer ZEN 3600, Malvern, UK) with a scattering angle of 90° at 25°C. The results were the mean values of three experiments for the same sample.

### Determination of drug loading content and encapsulation efficiency

In order to determine drug LC (w/w%) of micelles, propofol-loaded mixed micelles solution was freeze-dried and then dissolved in methanol and propofol content in micelles was determined on a Shimadzu series HPLC system (Shimadzu LC-10AT, Kyoto, Japan) equipped with a UV detector (Shimadzu SPD-10A) and reversed phase column (ODS C18, 5 μm, 4.6 mm × 250 mm, Dikma, China). The mobile phase consisted of acetonitrile/water (80/20, v/v) and was pumped at a flow rate of 1.0 mL/min. The detection wavelength was 270 nm. The LC of the micelles was then calculated based on the following formula:

To determine the propofol concentration in the aqueous phase of the micelles and CLE, separation of the two phases was performed using ultracentrifugation. Ultracentrifugation was performed at 10,000 rpm (14000 *g*) for 20 min. Amicon ultra centrifugal filter units (Amicon ultracel 4 k, Millipore, USA) were filled with 400 μL propofol samples and after centrifugation the aqueous phase was analyzed by HPLC as above. The encapsulation efficiency (EE) of the micelles was calculated based on the following formula:

### In vitro release of propofol from micelles

One milliliter of micelles solution with known propofol content was placed into a dialysis bag with molecular weight cutoff of 3 kD. The dialysis bag was immersed into a flask containing 30 mL of release medium (phosphate buffer saline (PBS), pH 7.4) containing 30% (v/v) alcohol (sink condition) which was kept in a constant temperature shaking water bath at 37°C and 100 rpm. At predetermined time intervals, aliquots (1 mL) of the release medium was taken and replaced by fresh medium. The content of propofol in the medium was measured by HPLC method as described above. The cumulative release percentage of propofol was calculated. The CLE was also tested as control.

### Stability of mixed micelles in bovine serum albumin solution

The stability of mixed micelles in bovine serum albumin (BSA) solution was assessed from the change of particle size of micelles and the micellar propofol upon incubation of the mixed micelles with 0.2% BSA solution [24,25]. The mixture was incubated under shaking at the speed of 100 rpm at 37°C for 24 h and determined the particle size at time interval, defined as *d*_i_. The average diameter of micelles before BSA treatment, *d*_0_, was also measured. The ratio of particle sizes was calculated as *d*_i_/*d*_0_. In addition, the determination of the EE was performed using the same method described above.

### Stability of mixed micelles under storage condition

The physical stability of mixed micelles under storage conditions was also evaluated. Freshly prepared drug-loaded mixed micelles were transferred into glass vials and then stored at 25°C for 6 months. The stability of micelles was monitored by the time-dependent changes in the physical characteristics, like drug precipitation, changes in micelle size, and drug LC during the storage period.

### Sleep/recovery studies

Male SD rats weighing 200 ± 20 g were used for this research. All animals were housed with free access standard food and tap water, and exposed to alternating 12 h periods of light and darkness. Temperature and relative humidity were maintained at 25°C and 50%, respectively. After an acclimatization period of 2 days, the rats were fasted for 12 h but allowed free access to water prior to the experiments. Eighteen rats were randomly divided using a random number generator into two groups (*n *= 9). The rats in each group received their respective formulations (mixed micelles sterilized by filtration through a 200-nm pore filter or CLE) via the caudal vein at a single dose of 10 mg/kg. The end of injection was taken as time zero (*t *= 0). After each administration, the time to loss of righting reflex was recorded for each animal. Rats were maintained in dorsal or lateral recumbency during evaluation, and the time of righting reflex return was recorded.

### Statistical analysis

All data were expressed as mean standard deviation (SD) unless particularly outlined. The statistical significance of differences among more than two groups was determined by one-way ANOVA by the software SPSS 13.0. A value of *p *< 0.05 was considered to be significant.

## Results and discussion

### Effect of Solutol HS15 content on critical micelle concentration of PEG5000-PLA4800

Polymeric micelles can be formed only when the block copolymer concentration is higher than CMC which characterizes the micelle stability [26]. One main concern following the use of polymeric micelles for drug carriers is the severe dilution they undergo in the biological environment (sometimes below the CMC). Therefore, the *in vitro *and *in vivo *stabilities of micelles depend upon CMC values of micelle-forming materials, and the CMC is an effective parameter of micelle preparation. Table 1 showed the results of influence of Solutol HS15 content on the CMC of mPEG5000-PLA4800. The CMC was 5.1 mg/L at 25°C for mPEG5000-PLA4800 diblock copolymer, indicating that the plain micelles might be stable *in vitro*. The trend that the CMC of mPEG5000-PLA4800 increased with the increase of Solutol HS15 content was observed, which was in accordance with our previous report [13]. This might be attributed to the reduction of hydrophobic interaction between the hydrophobic segments. It is well known that the stronger the hydrophobic interaction between the hydrophobic segments the lower the CMC of micelle-forming material [27,28]. In the case of our experiment, the hydrophobic segments forming the core of plain micelles and mixed micelles were PLA chains, and PLA and octadecyl chains, respectively. The hydrophobic interaction between PLA segments was greater than that between PLA segment and octadecyl moiety of Solutol HS15 molecules due to the high lipophilic character of PLA, and the more the content of Solutol HS15 the weaker the hydrophobic interaction [29,30]. Unfavorably, this increase in CMC suggested that the stability of mixed micelles toward dilution might be decreased compared with plain micelles formed by mPEG-PLA.

**Table 1:** Effect of Solutol HS15 content on the properties of the copolymer and polymeric micelle at 25°C.

Mole ratio of mPEG-PLA to Solutol HS 15	3:0	3:1	1:1	1:3	0:3
CMC (mg/L)	5.1	7.6	11.5	13.9	29.1

On the other hand, it should be noted that the CMC of mixed micelles decreased with increase of mPEG-PLA content. Clearly, a major problem encountered with Solutol HS15 plain micelles was their relatively high critical micelle concentration (Table 1), which resulted in the dissociation of micelles into monomers upon dilution. Due to this instability, additional stabilization was required by addition of mPEG-PLA. It was found that the CMC of the mixed system composed of mPEG-PLA and Solutol HS 15 at molar ratio of 1:1 was still lower (11.5 mg/L, expressed with the content of mPEG-PLA), which pointed on their high stability to maintain the integrity even upon strong dilution *in vivo *due to the fact that the content of mPEG-PLA in the prepared mixed micelles was almost 6 mg/mL.

### Optimization of micelle composition and characterization of mixed micelles

Drug-free plain and mixed micelles of mPEG-PLA/Solutol HS15 were prepared by rotary evaporation method, and propofol-loaded plain and mixed micelles were prepared by simply mixing drug-free plain and mixed micelles and propofol, respectively. Owing to the fact that size and size distribution might play a key role in determining the fate of micelles after administration, the effects of the addition of Solutol HS15 on the properties of plain micelles were evaluated. As shown in Table 2, micelles with nanoscaled size in diameter and narrow distribution were successfully prepared. Moreover, the micelle size appeared to be dependent on both copolymer composition and drug-loading. Similar to the paclitaxel-loaded polymeric micelles we reported earlier [13], the diameter of drug-loaded micelles was significantly larger than that of drug-free micelles. In addition, insertion of Solutol HS15 into plain micelles led to decrease in particle size, and there was a negative correlation between the size of mixed micelles and the content of Solutol HS15. These might be attributed to the fact that the chain length of octadecyl present in Solutol HS15 was shorter than that of PLA segment. It was reported that the size of polymeric micelles mainly depend on the chain length of hydrophobic segment [27,31,32]. More importantly, the LC of propofol in plain micelles was up to about 21%, which was highly significantly greater than that reported earlier [8] (the maximum LC was 12%) in which propofol was solubilized in PVP-PLA polymeric micelles. This might be mainly accounted for the micelle-forming materials with different hydrophilic block. In the case of our experiment, propofol was oriented with the nonpolar part of the molecule directed toward the core of mPEG-PLA micelles by hydrophobic interaction and the polar group toward the hydrophilic PEG chains through hydrogen bond between phenolic hydroxyl groups of propofol and ether oxygen of PEG [33,34], whereas propofol was only solubilized in micelles by hydrophobic interactions for the report mentioned above. In addition, the different preparation methods for micelles led to the different LC of micelles [27]. Noticeably, the LC of propofol in micelles increased with increase of Solutol HS15 content in mixed micelles (Table 2). When the molar ratio of Solutol HS15 to mPEG-PLA was 1:9, LC increased slightly compared with that of plain micelles (*p >*0.05), however, LC of mixed micelles with molar ratio of 5:5 for Solutol HS 15 to mPEG-PLA was significantly higher than that of plain micelles (*p *< 0.01), likely due to the fact that the interaction between octadecyl and propofol was stronger than that between PLA and propofol, or the compatibility between the core of mixed micelles and propofol molecules (the difference of partial solubility parameter between them was 1.7 (J/cm^3^)^1/2^) was better than that between the core of plain micelles and propofol molecules (the difference of partial solubility parameter between them was 2.3 (J/cm^3^)^1/2^) [35]. Moreover, there was no significant difference in LC between mixed micelles with 5:5 and 7:3 molar ratios of Solutol HS 15 to mPEG-PLA (*p *> 0.05). The optimal molar ratio of Solutol HS 15 to mPEG-PLA, i.e., 5:5 (equivalent to weight ratio of approximately 10:1), was therefore selected.

**Table 2:** The effect of molar ratio of Solutol HS15 to mPEG-PLA on properties of micelles.

Molar ratio of Solutol HS15 to mPEG-PLA	0:10	1:9	5:5	7:3
Drug-free micelles size (nm)	88.1 ± 4.1	83.3 ± 3.5	80.2 ± 3.6	78.3 ± 5.1
PDI	0.16 ± 0.03	0.17 ± 0.04	0.19 ± 0.07	0.16 ± 0.06
Drug-loaded micelles size (nm)	119.9 ± 5.5	117.1 ± 6.1	101.0 ± 3.8	94.4 ± 6.4
PDI	0.17 ± 0.11	0.19 ± 0.04	0.12 ± 0.09	0.18 ± 0.08
LC (%)	21.1 ± 3.2	26.5 ± 1.5*	32.4 ± 1.3**	36.0 ± 2.4***

* *p *> 0.05 versus plain micelles

** *p *< 0.01 versus plain micelles

*** *p *> 0.05 versus mixed micelles with 5:5 molar ratio of Solutol HS15 to mPEG-PLA

**Figure 1:**
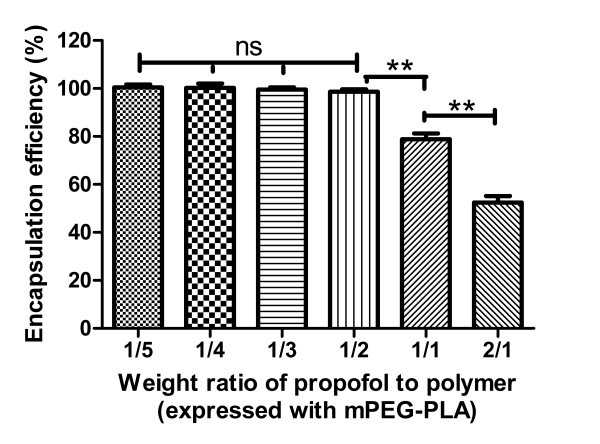
**The effect of the weight ratio of propofol to polymer (expressed with mPEG-PLA) on encapsulation efficiency (EE)**. ns: *p *> 0.05 between any two groups; ** *p *< 0.01.

To find an optimal ratio of propofol to mPEG-PLA and Solutol HS15, which allowed for the best propofol solubilization, a series of propofol-loaded mixed micelles was prepared with different weight ratios of propofol to polymer (1/5, 1/4, 1/3, 1/2, 1/1, 2/1, expressed with mPEG-PLA) at the same molar ratio of Solutol HS15 to mPEG-PLA (5:5), and the propofol micellization efficiency by each of the micelle dispersions was determined. The effect of the weight ratio of propofol to mPEG-PLA on propofol micellization efficiency was shown in Figure 1. It was clearly observed that there was no significant change in propofol micellization efficiency when the weight ratio of propofol to mPEG-PLA was lower than 0.5, suggesting that to acquire the best propofol solubilization, the ratio of mPEG-PLA/Solutol HS15/propofol was fixed at 10/1/5 (w/w/w), and the weight ratio of propofol to mPEG-PLA should be approx. 0.5.

**Figure 2:**
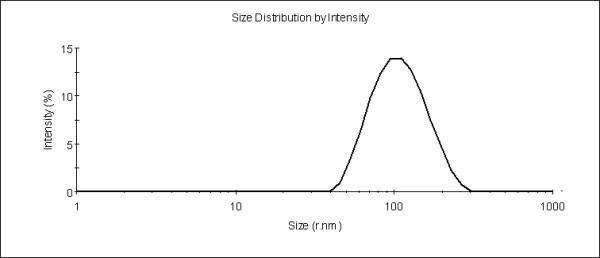
**Micelle size and size distribution of propofol-loaded mPEG-PLA/Solutol HS15 mixed micelles**.

Thus, our final optimized preparation of propofol-loaded mixed micelles had a composition as mPEG-PLA/Solutol HS15/propofol of 10/1/5 by weight, average micelle size of about 101 nm and PDI of 0.12 as shown in Figure 2. The LC of mixed micelles was about 32.4%, and the content of propofol in the preparation was approximately 0.3%.

### Determination of the content of free propofol in micelle solutions

**Figure 3:**
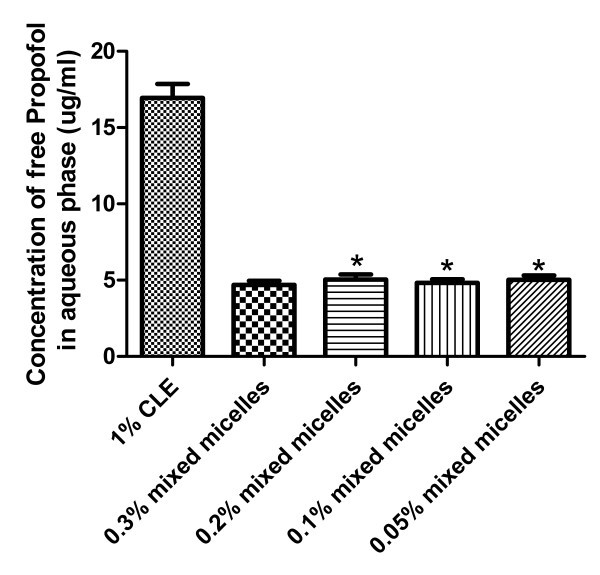
**Concentration of free propofol in the aqueous phase of the commercial lipid emulsion (CLE) and mixed micelle solutions**. Values are means ± SD (*n *= 3). * *p *> 0.05 vs. mixed micelles with 1% propofol.

It was reported that free propofol present in the aqueous phase (outside emulsion droplets and/or micelles) might be an important element causing pain on injection of propofol formulations [36-38]. The content of free propofol present in micellar solution and CLE was therefore determined, respectively. The samples were first separated into lipid (or micelles) and aqueous phases by ultrafiltration. As was seen in Figure 3, the concentration of free propofol in the aqueous phase of CLE containing 1% propofol was found to be 16.87 ± 1.06 μg/mL, whereas 4.69 ± 0.26 μg/mL in the mixed micelles solution with 0.3% propofol, indicating that the concentration of free propofol in mixed micelles was highly significantly lower (by about 72%) than that in CLE (*p *< 0.001) and that reported previously [39,40]. This might be accounted for by the fact that the micelles are thermodynamically stable system in which the solubilized drug tends to be embed inside micelles until the micelles decompose, just like the results presented in Table 3, whereas emulsion (CLE) is thermodynamically instable system, partial phase inversion or separation led to the leakage of the drug. In addition, there was no significant change in the concentration of free propofol in micelle solution upon dilution (Figure 3), whereas CLE exhibited phase separation upon this stronger dilution. These suggested that propofol-loaded mixed micelles might reduce the incidence and intensity of pain on injection of propofol as compared with CLE and therefore might have favorable compliance to the patients.

**Table 3:** The stability of mixed micelle at room temperature (25°C).

	0 month	6 month
Particle size (nm)	101.3 ± 5.3	103.6 ± 6.2
PDI	0.16 ± 0.06	0.18 ± 0.05
Concentration of free propofol (μg/mL)	5.28 ± 0.38	5.19 ± 0.52
LC (%)	35.3 ± 2.8	33.7 ± 2.5

### Stability of mixed micelles

**Figure 4:**
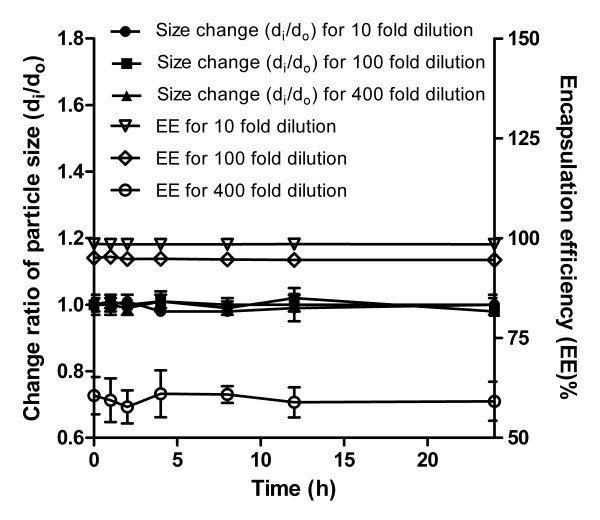
**Changes in particle size and encapsulation efficiency (EE) of propofol-loaded mixed micelles in 0.2% BSA solution at 37 °C at different dilution extent (*n *= 3)**.

A basic evaluation of pharmacokinetic modeling and efficacy data for micelles or drug loaded micelles in animal models was conducted, testing the stability of drug loaded micelles in the presence of serum or serum albumins [41]. As shown in Figure 4, the ratio (*d*_i_/*d*_0_) of mixed micelles did not change significantly at different dilution extent within 24 h, indicating that mPEG efficiently protected the BSA adsorption, which resulted from the hydrophobical interaction of the hydrophobic groups with BSA, on the mixed micelles because the hydrophobic groups of the copolymer were efficiently screened by mPEG [42].

Additionally, the micellar propofol was also determined to further elucidate the effect of dilution on stability of mixed micelles. The results showed no significant change in EE of mixed micelles for 10- and 100-fold dilution (Figure 4), whereas the mixed micelles presented dramatic change in EE under 400-fold dilution, suggesting that the mixed micelles were stable in the presence of BSA at lower dilution extent, and part of the micelles began to dissociate when they were diluted near to critical micellization concentration. Nevertheless, the concentration of free propofol in aqueous phase was only in the range 1.2 to 5.0 μg/mL, which was considerably lower compared to the CLE, due to the huge dilution extent.

The storage stability of mixed micelles was also tested at 25°C for 6 months. As shown in Table 3, propofol-loaded mixed micelle formulations did not show any noticeable change in the particle size and PDI, and no precipitation of drug was found during this period. In addition, the LC of mixed micelles had a loss of only 2.4%, probably due to the partial decomposition of propofol. These indicated that the drug-loaded micelles were physically stable at room temperature for at least 6 months. The long-term stability of propofol-loaded mixed micelles is currently being evaluated.

### *In vitro *release of propofol from micelles

**Figure 5:**
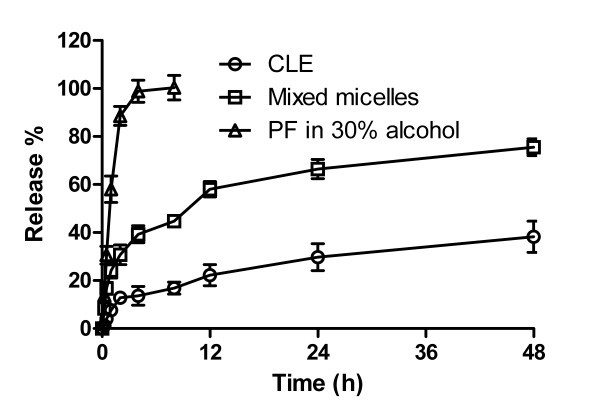
**Release profile of propofol from mixed micelles, the commercial lipid emulsion (CLE) and 30% w/w alcohol solution at 37°C**.

When developing intravenous colloidal delivery systems for highly hydrophobic drugs such as propofol, it is important to adequately control the release rate in order to avoid precipitation upon dilution in blood. The *in vitro *release of propofol from mixed micelles was, therefore, investigated. Prior to conducting this release assay, it was verified that propofol could freely diffuse through the dialysis membrane (Milipore Co. Ltd, USA) when the molecular weight cut-off of the membrane was 3 kD (Figure 5). Moreover, the sink condition was respected by addition of 30% (v/v) ethanol in the release medium. More importantly, no significant change in micelle size upon incubation of the micelle sample with release medium at 37°C with time from 0 to 24 h was observed (data not shown), indicating that the integrity of propofol-loaded mixed micelles was not affected by the release medium upon 24 h long co-incubation.

The propofol release profile from mPEG-PLA/Solutol HS15 mixed micelles was presented in Figure 5. The comparison of the profiles of propofol release from the micelles and the aqueous solution showed that the entrapment of propofol in the nanoparticles could significantly retard its *in vitro *release [11,18,23]. In addition, it was observed that the propofol release from mPEG-PLA/Solutol HS15 mixed micelles started with an initial burst, followed by a very slow release phase. This release behavior could be explained through the geometry of propofol location in the micelles and reflected the propofol incorporation stability. The initial burst happened within the first 4 h, especially within the first 2 h, was mainly attributed to the drug located in the hydrophilic corona or shell of micelles, and could proceed via both the hydration of the interfacial drug molecules and their passive diffusion. Thereafter, the slower propofol release resulted from that localized in the inner core of micelles.

In addition, the cumulative release percentage, about 75.5%, of propofol from mixed micelles within 48 h was about 2-fold greater than that (about 38.3%) from commercial formulation (CLE), and the accumulative release of mixed micelles was significantly higher than that of CLE at all the time points. These might be accounted for the larger globule size of CLE induced relatively slower diffusion of the drug from inner phase and then slower release. Nevertheless, the release rate of mixed micelles was faster than that of CLE before the first 12 h, the release rate of the two formulations was comparable thereafter. Overall, quick release rate for mixed micelles might produce a favorable pharmacological effect for drugs, especially for propofol, which exhibits rapid sleep and recovery.

### Sleep/recovery studies

**Figure 6:**
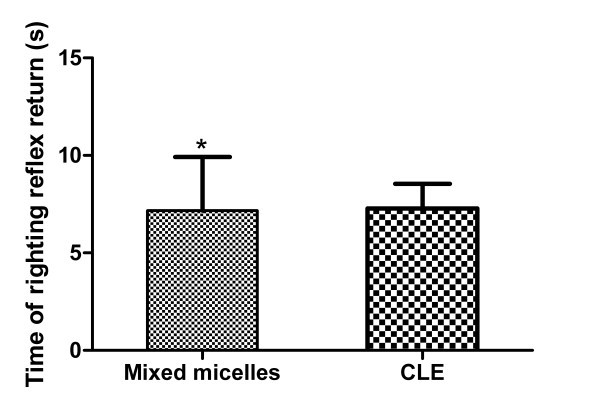
**Sleep-recovery study results**. The dose was 10 mg/kg (onset of sleep was less than 0.5 min). * *p *> 0.05 vs. the commercial lipid emulsion (CLE).

The mean time values to loss and recovery of righting reflex were evaluated for mixed micelles and the CLE. It was found that animals rapidly lost righting reflex (within 0.5 min of dose administration) following administration of both of propofol formulations (data not shown). The results of recovery of righting reflex were presented in Figure 6. Time for righting reflex recovery was 7.17 ± 2.75 and 7.29 ± 1.25 min, for mixed micelles and CLE, respectively. On average, both groups of animals given propofol formulations recover their righting reflex after 7.2 min. It was concluded that the time for the animals to lose and recover righting reflex was essentially the same for both formulations of propofol (*p *> 0.05). In addition, no animals sustained any observable toxic effects from use of either propofol-loaded mixed micelles or CLE. Results from this pharmacological paradigm in rats suggested that the mixed micelles had very similar pharmacological effects as CLE. Based on these data, it might be inferred that the difference in propofol release from the two formulations did not significantly affect the partition of the drug to the site of action (e.g., the central nervous system) in rats, hence explaining the similarity in the pharmacological effects observed.

## Conclusions

Mixed micelles consisted of mPEG-PLA and Solutol HS15 were designed and provided a more efficient solubilization of the poorly soluble drug, propofol, as compared with plain micelles made of mPEG-PLA alone. Stability studies showed that the mixed micelles were stable in the presence of BSA at different dilution extent and there was no effect of dilution on the stability of micelles, which is very important for a drug delivery system because one of the important differences between the *in vitro *and *in vivo *conditions is the dilution effect under *in vivo *administration. In addition, the drug-loaded mixed micelles were physically stable at room temperature for at least 6 months. As anticipated, the free propofol present in the aqueous phase was significantly reduced by mixed micelles. The pharmacological effect of mixed micelles was proved to be comparable to that of the commercial formulation. This study not only provided an idea for preparing a novel drug carrier from two amphiphilic materials, but also overcame some drawbacks on propofol injection.

## Abbreviations

BSA: bovine serum albumin; CLE: commercial lipid emulsion; CMC: critical micellization concentration; DLS: dynamic light scattering; EE: encapsulation efficiency; LC: loading content; mPEG-PLA: poly(ethylene glycol)-poly(lactide); PBS: phosphate buffer saline; PVP-PLA: poly(*N*-vinyl-2-pyrrolidone)-block-poly(d,l-lactide); SD: mean standard deviation.

## Competing interests

The authors declare that they have no competing interests.

## Authors' contributions

QZ and YL conceived of the study and involved in revising the manuscript critically. XL carried design and coordination of study, anticipated all of experiments, and wrote the manuscript. YZ participated in the synthesis of mPEG-PLA, carried the HPLC experiments and contributed to data interpretation. YF performed the pharmacological experiments, organized the results. XW and CF performed the release and stability measurements. YZ participated in the experimental setup development, discussions and data analysis. All authors read and approved the final manuscript.
